# Amplifying Domain Expertise in Clinical Data Pipelines

**DOI:** 10.2196/19612

**Published:** 2020-11-05

**Authors:** Protiva Rahman, Arnab Nandi, Courtney Hebert

**Affiliations:** 1 The Ohio State University Columbus, OH United States

**Keywords:** review, data analysis, data science, clinical informatics

## Abstract

Digitization of health records has allowed the health care domain to adopt data-driven algorithms for decision support. There are multiple people involved in this process: a data engineer who processes and restructures the data, a data scientist who develops statistical models, and a domain expert who informs the design of the data pipeline and consumes its results for decision support. Although there are multiple data interaction tools for data scientists, few exist to allow domain experts to interact with data meaningfully. Designing systems for domain experts requires careful thought because they have different needs and characteristics from other end users. There should be an increased emphasis on the system to optimize the experts’ interaction by directing them to high-impact data tasks and reducing the total task completion time. We refer to this optimization as amplifying domain expertise. Although there is active research in making machine learning models more explainable and usable, it focuses on the final outputs of the model. However, in the clinical domain, expert involvement is needed at every pipeline step: curation, cleaning, and analysis. To this end, we review literature from the database, human-computer information, and visualization communities to demonstrate the challenges and solutions at each of the data pipeline stages. Next, we present a taxonomy of expertise amplification, which can be applied when building systems for domain experts. This includes summarization, guidance, interaction, and acceleration. Finally, we demonstrate the use of our taxonomy with a case study.

## Introduction

Recent advancements in data availability (eg, digitization of health records) and deep neural networks [1] have led to the resurgence of artificial intelligence. This has served as a catalyst for data-driven decision making in many domains. However, for high-stakes applications, such as financial and health care domains, it is rare for domain experts to execute decisions solely based on artificial intelligence algorithms [2]. Domain experts in this context are individuals who are not necessarily trained in computational fields but inform the design and are end users of data-driven algorithms (eg, health care providers, hospital administrators). Note that domain experts can have different levels of expertise in their specific domain (eg, interns, residents, attendings), and we do not differentiate between these levels in this work. Although the role of experts has been studied in clinical decision support (CDS), we find a gap in their involvement in the data analysis pipeline, which we focus on in this work.

**Figure 1:**
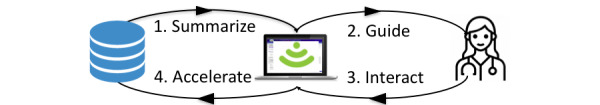
Domain expertise amplification.

Domain expert involvement remains necessary in the health care domain, but this involvement brings significant challenges and implications for data-driven applications. Domain experts are expensive resources with limited time for these efforts, and excessive reliance on domain expertise could potentially lead to systems that are overly customized and not reproducible or scalable. Owing to these challenges, designing systems for them requires careful consideration. To address these challenges, we present a framework for amplified intelligence that identifies the points in the process where expertise can be effectively leveraged. *Amplification of expertise* then refers to the process of automating redundant or inferable tasks, so that domain experts can focus their efforts on tasks that require domain knowledge. This is a synergy between the domain expert and the system, which involves summarization of data and decisions, guidance toward insights, interaction by the domain expert, and acceleration of input (Figure 1).

### Prior Work

There is active research on interactive and human-in-the-loop systems in many computer science disciplines. The database and visualization communities have produced numerous tools [3-8] to aid data scientists with data wrangling and analysis. At the decision-making stage, the machine learning community has looked at making black box models explainable [2,9-12], while the human-computer interaction (HCI) community has been studying how differences in explainability affect decision making [13,14]. Finally, the crowdsourcing community has concentrated on human-powered computation by optimizing tasks (eg, simplifying tasks [15], minimizing the number of questions [16,17], optimizing workflows [18-20]). However, we focus on data-powered experts by amplifying expertise. Although we draw from prior work, systems designed for health care domain experts require special consideration because they have characteristics that distinguish them from data scientists and crowdworkers.

### Special Considerations in the Health Care Domain

First, domain expert input is usually needed for data tasks that require experiential knowledge and judgment (such as medical diagnoses and forensic analysis [21]). The critical and subjective nature of these decisions necessitates transparency, both from the algorithm and domain experts. Hence, the system needs to summarize the impact of algorithmic or experts’ manipulation of the data [22]. Second, due to their specialized training, domain experts’ time is expensive and limited [23,24]. This constraint makes it imperative that we build tools that provide insights while reducing physical and cognitive effort [25]. Third, as domain experts are trained in noncomputational fields, systems designed for them should provide high-level interaction capabilities. This is referred to as *editable shared representations* between computers and humans [26]. Examples include natural language interfaces and form-based input [27]. Finally, domain experts are highly trained individuals, which allows systems to accelerate their input by using domain-specific assumptions and ontologies [28,29]. Keeping these factors in mind, expertise amplification involves summarization, guidance, interaction, and acceleration (Figure 1). We will explore each of these in detail in the following sections.

### The Data Pipeline

There are opportunities to amplify expertise at all stages of the pipeline. The data pipeline refers to the different stages that the data need to go through before they can provide decision support. It can roughly be broken into 3 stages: curation, cleaning, and analysis. Tools at the end of the pipeline have only looked at explaining models but not at amplification. In contrast, tools at earlier pipeline stages have been designed mainly for data scientists and not for experts. However, domain experts are involved at every stage of the pipeline [27-31], especially in clinical research settings where data sets contain specialized information. Thus, there is a need to amplify domain expertise throughout the pipeline. In this work, we provide examples from the informatics literature to highlight the need for expert involvement at each pipeline step. We then review literature from the database, HCI, and visualization communities about challenges and current approaches at different stages. On the basis of our review, we present a novel taxonomy for amplifying domain expertise and demonstrate its use with a case study in empiric antibiotic treatment. Our review can serve as a guide to new clinical research projects, and our taxonomy can be applied when designing systems for experts, especially for low-budget projects when there are limited resources and availability of domain experts.

## Challenges in the Data Pipeline

This section is organized to reflect the clinical data pipeline, which often involves the following steps: data are curated from the electronic health record (EHR) data warehouse and annotated with external data sources, cleaned and validated, and analyzed. Multiple people are involved in various stages of the pipeline. The prevalent notion of the workflow is that a data engineer restructures, cleans, and sets up the infrastructure for data analysis, and a data scientist then analyzes and models the data, which a software engineer implements into a decision support system. A domain expert then consumes the end product to make decisions. However, in clinical settings, domain expert involvement is required at every step of the pipeline. Allowing domain experts to directly and efficiently interact with data removes the need for them to rely on a data engineer or data scientist who can then focus on infrastructure and model construction. Moreover, since domain experts are the stakeholders in the output of data pipelines, in our experience, they tend to be engaged users who want to interact with data and leverage their expertise. In this section, we motivate domain expert involvement with examples from the past five years of research presented at the American Medical Informatics Association’s annual symposiums. We then review the computer science literature to identify current tools and opportunities for expertise amplification at the 3 stages of the data pipeline: data curation, data cleaning, and data analysis, as each of these corresponds to a research area of its own.

### Data Curation

Curating data sets for analysis can be a laborious process that can involve combining multiple data sources and identifying relevant attributes. Data integration and data discovery address these problems.

#### Data Integration

Medical data pipelines often involve data that were collected for purposes other than answering the research question at hand. This usually implies that information is not captured in a manner fit for analysis [32,33], with issues such as missing metadata information [34]. Moreover, in some situations such as rare disease studies, the cohort size is too small for analysis [35], while in other cases, external features such as air quality or drug components [36-39] might be needed. One possible solution to these data quality issues is to curate data from multiple institutions and external sources. However, the different data representations [35,40] pose challenges in entity matching, metadata inference, and data integrity [41,42]. Data integration aims to automatically resolve schema matching and entity matching problems during data curation. For biomedical data sets, integration can involve standardization by mapping to ontologies with controlled vocabularies [43-45]. Although current approaches use deep learning for integration [46-50], generating a training corpus and validating results require domain expert input. For example, Cui et al [35] require domain experts to validate data curation efforts for studying sudden death in epilepsy. In another example, building an automatic concept annotator for standardizing biomedical literature [50] required experts to manually annotate different concepts [51-54]. Furthermore, a domain expert will be able to catch inconsistencies or errors made by an automated integration tool much faster than a data engineer who is unfamiliar with the domain. Thus, there is a need to build interactive data integration tools for domain experts.

#### Data Discovery

Data discovery refers to the process of finding relevant attributes or cohorts for analysis. This is especially true for multidisciplinary teams where the domain expert knows the disease definition but is not familiar with the database schema. At the same time, the data engineer can explore the schema but might not recognize that a field is relevant. Integrating data from multiple sources only exacerbates this problem. In the informatics community, DIVA [55] aids in cohort discovery by ingesting expert-defined constraints, while visual analytic systems [56,57] such as CAVA provide an interactive interface. In the database community, Nargesian et al [58,59] have looked at finding unionable (more data points) and joinable (more attributes) data for a given data set. These algorithms are useful when trying to augment data sets with publicly available data sets such as MIMIC [60] or even for exploring a complex schema such as the Unified Medical Language System (UMLS) [61]. In addition to using properties of the data to find possible attribute matches, domain rules can be useful for identifying relevant data subsets. This requires an interactive interface where domain experts can look at subsets of interest and iteratively join and filter the data [62] to find the required cohort. Recently, query logs have been used to design precision interfaces [63,64] that customize the interface for the user’s task.

### Data Cleaning

After curating relevant data sets, data still need to go through multiple preprocessing steps before they are analysis-ready. These include identifying and fixing incorrect data, data augmentation, and data transformation [65], all of which benefit from domain expert involvement.

#### Error Fixes

EHR data are known to be messy and have errors and missing values [66-68]. A typical data cleaning method is the use of rule-based systems that identify dirty data by detecting violations of user-specified rules or known functional dependencies [69-78]. These systems do not optimize the expert’s rule specification process. Crowdsourcing systems have also been used to correct values [18,79], although they are not always an option due to data complexity or confidentiality. Another approach to identify and clean data is to augment the data with external knowledge bases [80-82]. More recently, there have been many approaches [83-85] that use deep learning for automated data cleaning. Of note is Holoclean [84], which uses a statistical model to combine various data repair signals such as violation of integrity constraints, functional dependencies, and knowledge bases. Although this achieves higher performance than using each method in isolation, there is scope for identifying which of the signals are performing the poorest or what additional information would help improve the system’s performance. Identifying this information, incorporating domain knowledge, and presenting it succinctly to a domain expert remain open problems.

#### Data Augmentation

Although data entry errors [86] and missing information can be imputed by semiautomated methods, a more difficult problem is that of creating a gold standard for training data, which is referred to as data augmentation. Many health care applications require annotating training data, for example, clinical text annotation [87-89], CDS [90-92], identifying new terms for ontologies [93], index terms for articles [94], and disease-specific annotations [51,95,96]. However, very few applications focus on optimizing the domain expert’s data augmentation effort, which is eventually crucial to model performance. A notable approach to this is the Snorkel system [97], which automates data augmentation by learning the labeling function, thus accelerating the domain expert’s input. However, there are opportunities to make the initial labeling process more interactive, as domain experts are required to write code in Snorkel. Furthermore, the system does not provide feedback on how labels affect the data set or final model, which is crucial for building trust in medical pipelines. Examples of interactive solutions include Icarus [28] for augmenting microbiology data and Halpern et al's system [98] for annotating clinical anchors. Both systems use an ontology to interactively amplify domain expertise.

#### Data Transformation

Other than fixing incorrect values and augmenting data sets, often, data need to be restructured (eg, splitting values in a column, reformatting dates). Data wrangling has emerged as a separate field in the past decade because of data diversity. Potter’s Wheel [99] is one of the first interactive data transformation systems. It allows the user to specify data transforms that are encoded as constraints and used to detect errors. Building on this idea, systems such as Polaris [100] and Trifacta [4,101] infer syntactic rules from user edits. Similarly, programming-by-example systems [102,103] learn transformations from a set of input-output pairs. These techniques have informed the autofill function of Microsoft Excel. As many domain experts employ Excel for data transformations and analysis [104], spreadsheet interfaces should consider incorporating domain knowledge.

### Data Analysis

We now move to the final step of the pipeline. This includes exploratory analysis to identify attributes of interest and explainability of models for decision making.

#### Data Exploration

During the exploration step, it is crucial for the domain expert to be able to directly interact with the data for effective hypothesis generation. However, domain experts often must go through a data engineer to execute the relevant query [105,106] or extract information from unstructured notes [107]. The data are then validated by the domain expert through manual chart review, since data engineers without domain knowledge may apply naive filters that hide insights or find spurious correlations. To address these challenges, the informatics community has built tools to accelerate chart review [108] and allow interactive filtering and analysis [109,110]. Finalizing an analysis data set can then take multiple iterations of requests and validations between the domain expert and data engineer. In some cases, data engineers create custom dashboards for domain experts [111-113], but the latter are then limited to brushing and linking on the provided view. Mixed-initiative interfaces such as Tableau [100] and Dive [5] recommend visualizations based on statistical properties of the data but do not use domain-specific ontologies that can enrich the domain experts’ interaction and accelerate their workflow.

Visualizations, when used appropriately, can provide effective summaries and reveal patterns not immediately evident by statistical overviews [114]. Summaries reduce the cognitive load on domain experts during multidimensional data exploration, allowing them to drill down to specific instances as needed [115]. Although many visualization recommendation systems exist for analyzing numerical data [7,116-118], visualizations in health care often include categorical and text data [119-122]. As such, node-link diagrams are a common data representation and have been used for tracking family history [123], decision making [22,124], and identifying hidden variables [125]. Visual interfaces thus amplify expertise by summarizing data. However, they can be more powerful if they allow interaction, provide guidance by highlighting interesting regions for exploration [126], and accelerate workflows by extrapolating domain expert interactions based on properties of the data [22]. Thus, there is a need to provide domain experts with tools that allow for more sophisticated data interaction.

#### Explainability

Finally, we cannot discuss clinical pipelines without discussing explainability. The interpretability of rule-based systems has made them popular in a variety of clinical applications, including decision support [127,128], antibiotic recommendation [129], updating annotations [130], and auditing [131]. Interpretability is essential because domain experts want a cause-and-effect relationship, based on which actionable decisions can be made [66,68,132]. Furthermore, health care providers may not use models they do not trust, and building trust requires providing context and explanations [2].

Current approaches in health care research use weights and activation of features to characterize attribute importance [133-135]. RuleMatrix [136] provides an alternate approach where a set of rules represents the deep learning model. The expert can explore various facets of each rule, such as data affected, distribution, and errors. In another example, Cai et al [29] built a tool to help pathologists find similar images to aid in diagnoses. The tool allows domain experts to search for similar images and then interactively refine the search results. It allows refinement by region (crop an image), refinement by concept (filter by extracted concepts from image embeddings), and refinement by example (select multiple images as examples). These refinement techniques are examples of acceleration, where interactions are interpolated to the entire data set by learning general functions. Explainability is thus key to the adoption of deep learning models. Although they have mainly been applied in the analysis stage of the pipeline, they are equally important when applying automated algorithms to curation and cleaning.

Therefore, amplifying domain experts’ abilities in the analysis stage requires interactive data systems using a combination of statistical algorithms and compelling visualizations. Moreover, these systems need to follow design-study principles [137]. They need to allow interaction with domain experts for a needs assessment and an empirical evaluation to ensure that correct information is portrayed effectively. Otherwise, the system can end up burdening and biasing the domain expert instead of helping [13,101].

We have highlighted the need for domain expert involvement in the pipeline and describe some of the challenges they encounter. Although we have briefly expanded on some available solutions, Table 1 provides a more comprehensive list of references. Summarizing each technique is outside the scope of this paper, but it provides a guide to interested readers for further reading.

**Table 1:** Review of current approaches for each data pipeline stage.

Current solutions			Domain expert role	
**Data curation**				
	**Data integration**			
		Schema matching [138-143] Interactive integration [144,145] Webtables integration [146-151] Machine learning [46-49]		Domain experts are needed to validate results of integration, and interactively correct automated methods, which can then update their algorithm
	**Data discovery**			
		Attribute search [58,59,152,153] Interactive querying [55,62-64] Visual analytics [56,57]		Domain expert feedback is needed to finalize the analysis data set
**Data cleaning**				
	**Error fixes**			
		Rule-based [69-77,154] Crowdbased [18,79,155,156] Knowledgebase [80-82] Machine learning [83-85] Functional dependency [15-23,25-54,58-165]		Domain expert input can be used to identify and fix errors
	**Augmentation**			
		Machine learning [97,166,167] Interactive [28,98,168-170]		Domain experts can augment missing data with domain-specific rules
	**Transformation**			
		Programming by example [102,103] Interactive rules [4,99-101] Foreign-key detection [153,171-175]		Domain experts can restructure the data to make it semantically valid
**Data analysis**				
	**Exploration**			
		Optimize performance [176-179] Optimize insight [126,180,181] Provenance [182,183] Visualizations [5,7,116-118,184-188]		Domain experts interact with summaries and outliers to draw insight
	**Explainable**			
		Systems [189,190] Visualizations [9,12,29,136] Empirical studies [10,11,13,14]		Domain experts inform the model design to ensure explainability

## Taxonomy of Expertise Amplification

**Figure 2:**
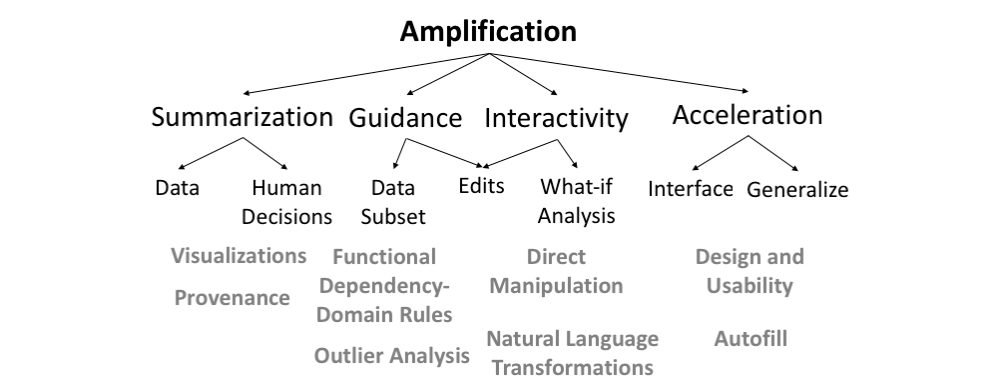
Taxonomy of expertise amplification: the first level shows the 4 dimensions that should be employed by a system for expertise amplification. The second level enumerates the subdimensions along which amplification can be done, while the fourth level in gray shows tools that can be applied.

The previous section elucidated the need for domain expert involvement throughout the clinical data pipeline. In all steps, domain expert involvement can improve automated methods but must be implemented appropriately to ensure that the process remains robust and reproducible. Taking this into consideration, we propose a taxonomy that can be employed when designing systems to amplify expertise in the clinical pipeline. Domain expertise amplification by a system can broadly be categorized into 4 dimensions: summarization, guidance, interactivity, and acceleration, as shown in Figures 1 and 2. Thus, a system that wishes to amplify expertise should apply one or more of these dimensions. We demonstrate these categories with examples from computer science literature.

### Summarization

The time constraints of experts along with transparency requirements in the clinical domain motivate the need for effective summaries of data and human decisions. Although data summaries are important for analysis, summaries of human decisions allow for improved explainability and reproducibility.

#### Data

An amplification system should summarize large and complex data sets so that experts can meaningfully consume them. This is relevant for identifying inconsistencies as well as for open-ended exploration during analysis. It can be overwhelming for an expert to go through large and wide tables. Therefore, amplification systems should automatically summarize complex data [191]. Although providing data samples [28,76] and statistical summaries such as mean, variance, and standard deviation can be useful for providing a bird’s eye view, they are not always enough to reveal patterns [114]. In such cases, *visual summaries* can provide additional insight, as done by the CAVA system [56]. Multidimensional data can be visually summarized by presenting each dimension as a coordinated histogram with linked brushing and filtering [176].

#### Human Decisions

In addition to data, amplification systems need to summarize algorithmic and human decisions as well. This is because domain expert involvement is usually required in situations where it is necessary to have high-quality data [2,21]. Hence, amplification systems also require high transparency [189,192]. To support algorithm transparency, amplification systems can show visual activation of features that led to the recommendation [9] or similar cases in the data that serve as evidence for the current recommendation [193]. Summarizing human decisions can involve expressing data transformations as natural language rules [4,28] and visual node-link diagrams [22]. Furthermore, as summarized data provide an abstract or aggregate view, there is a need for data transparency, meaning that experts should be able to trace individual data points, which contributed to the aggregate summary. This involves incorporating ideas from *provenance systems* such as Smoke [182] and Scorpion [183], which provide fast data lineage tracking. Finally, for each application, empirical studies are needed to see what and how information should be presented or summarized because too much transparency can overwhelm and negatively impact the expert [13].

### Guidance

Although summaries provide a global view of the data, the goals of exploratory analysis include finding insights and data quality issues [191], which might require looking at a more detailed view. Systems can guide experts by navigating to informative subsets and by suggesting data transformations and edits.

#### Data Subset

Amplification systems should guide the expert’s navigation to meaningful subsets. For example, SeeDB [116] automatically finds interesting visualizations. Given a query, it defines *interestingness* as the deviation of the query’s result set from a baseline data set. In a similar vein**,** TPFlow [194] uses tensor decomposition to guide users to interesting regions in spatiotemporal exploration. For data cleaning, error detection algorithms such as Uguide [78] and DataProf [76] use *functional dependencies* and Armstrong samples, respectively, to find incorrect tuples for human validation, while Icarus [28] presents the expert with impactful subsets for data completion. Visual summary tools such as Profiler [184] use statistics to find data quality issues. When guiding users with visual summaries, it is important to select optimal visual encodings to reveal relevant insights or *outliers*. This can be informed from recent work by Correll et al [185], which empirically evaluated different visual encodings on their effectiveness in revealing data quality issues.

#### Edits

In addition to navigating data sets, amplification systems can also guide experts by suggesting data transformations to edit the data during the cleaning and preparation stage [4,28,103]. However, even in this case, transparency is required. This is evidenced by the fact that in empirical studies of Proactive Wrangler [101], users often ignored the suggested transformation but then manually performed the same one because the semantics of the operation were unclear. Methods to aid in data transformation transparency include showing previews and transitions of the data changes [195] resulting from the transformation operation.

### Interaction

Along with making system internals explainable [10], allowing experts to interact and modify data and the output of algorithms increases their trust in amplification systems [11]. For empiric antibiotic recommendation [196], this can involve allowing the health care provider to edit model features. Providing interaction comes at the cost of maintaining strict latency constraints since experts expect to see the results of interaction almost immediately [137]. Techniques for maintaining interactive performance include sampling [197] and predictive prefetching [198]. Interaction modes can include data transformation suggestions and what-if analysis.

#### Data Transformation

The mode of interaction for data transformation in expertise amplification systems also needs to cater to their background and training. For example, transformations should be presented as *natural language* statements [4] as opposed to code snippets [97,154]. Although graphical user interfaces can decrease trust and control for system administrators [199], they are needed in amplification systems. Gestural query systems, such as GestureDB [62] and DBTouch [200], and *direct manipulation interfaces* might be preferable to domain experts who are unfamiliar with SQL. Furthermore, domain experts’ affinity for spreadsheet tools [104] motivates designing systems with spreadsheet interfaces but advanced querying capabilities such as Dataspread [201] and Sieuferd [202].

#### What-if Analysis

To support collaborative decision making, amplification systems should allow for what-if analysis, where domain experts can apply or test different *decisions* and *assumptions* and see how it affects the data set. Collaborative decision making is important for consensus and conflict resolution. Domain experts are highly trained and experienced individuals in their fields, which affects how they interact with systems [203,204]. Data pipeline tasks that require their input need them to apply knowledge from training and experience [28]. Such tasks inherently require judgment, which can be biased and can vary between and within domain experts [205]. To account for this bias, consensus from multiple experts is needed. However, unlike crowdworkers, where differences in results can indicate bad actors entering random choices [18,206,207], in the case of domain experts, they reveal differing judgments. As such, automatic conflict resolution [208], such as majority voting, cannot be used because disagreements require expert discussion [22]. Collaboration is required for conflict resolution, and what-if analysis can speed up this process. Capturing and sharing metadata is also useful for collaboration [209-212].

### Acceleration

Time constraints of domain experts necessitate the need to accelerate their input provision. This involves designing interfaces that aid the expert’s task and building interactions that interpolate from edits to generalize to multiple data points.

#### Interface Design

Most experts use structured interfaces such as forms [213] or free-text notes [214] for data entry or querying and spreadsheet interfaces for data exploration [104]. Following *user-centered interface* design and adhering to latency constraints is even more essential for these systems. Query interface layouts can be optimized by using statistical properties of the data [215-217] and prior query logs [64,218], while spreadsheet interfaces can be improved by incorporating higher expressibility [201,202]. The Usher [216] system, an example of the former, uses a probabilistic model on prior input form data to optimize the form structure. This involves showing highly selective data attributes at the beginning of the form to reduce the complexity at later stages, thus reducing the scope of error and accelerating input provision.

#### Generalize

An advantage of building systems for domain experts is that domain-specific information can be used to accelerate their input. For example, Icarus [28] uses the organism and antibiotic hierarchy encoded as foreign-key relations in the database to generalize a single edit to a rule that fills in multiple cells, accelerating the data completion process. In another example, the system by Cai et al [29] allows domain experts to refine result sets with domain-specific concepts extracted from image embeddings.

## Case Study

**Figure 3:**
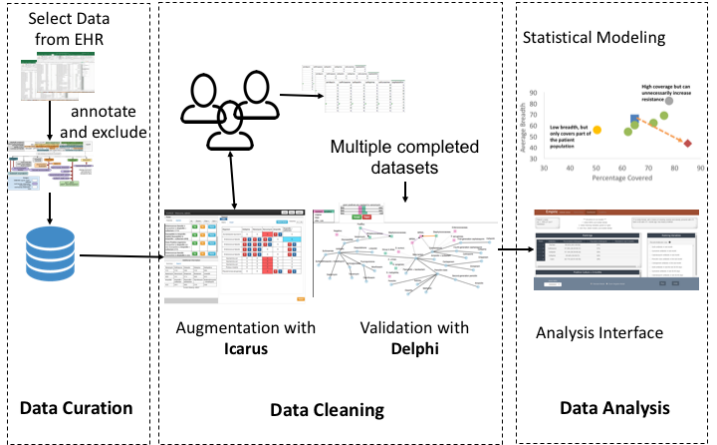
Data pipeline for empiric antibiotic prediction. EHR: electronic health record.

We illustrate our taxonomy with a case study from a representative clinical data project: modeling empiric antibiotic treatment (Figure 3). We apply the 4 dimensions of amplification to the 3 stages of the pipeline. This is summarized in Table 2.

**Table 2:** Applying 4 dimensions of amplification to the clinical data pipeline for empiric antibiotic prediction.

Domain expert task				Amplification	
**Data curation**					
	Identify variables of interest, validate patients included in the cohort, and make domain-specific exclusionary rules			*Summarization*: present distribution of variables of interest *Guidance*: suggesting additional variables based on the selected ones *Interactions*: allow expert to select and remove data points *Acceleration*: suggest criteria based on the domain expert’s inclusion and exclusion	
**Data cleaning**					
		**Augmentation**			
			Fill in unreported microbiology susceptibilities with rules		*Summarization*: preview a rule by showing distribution of the cells that will be impacted *Guidance*: show high-impact data subsets for edits *Interactions*: direct edits on interface and indirect edits via rules *Acceleration*: suggest general rules based on the domain expert’s single edit
		**Validation**			
			Validate data augmentation by examining rule set and consolidating them to remove conflicts		*Summarization*: visual summary of rules and their relations *Guidance*: node size guides user to high-conflict areas *Interactions*: edit rule set by accepting and rejecting rules *Acceleration*: automatically remove redundant rules
**Data analysis**					
	Understand the model and its predictions for individuals and different patient subpopulations			*Summarization*: show probability of coverage with confidence interval *Guidance*: highlight covariates of concern *Interactions*: allow domain expert to select covariates to include *Acceleration*: show similar patients for who the model should be updated	

At the data curation level, our domain expert, Lucy, must provide the cohort definition along with variables of interest (eg, demographics, comorbidities, allergies, etc) to a data engineer, who pulls the relevant data from the EHR data warehouse. After the data pull, Lucy looks through the initial set and formulates additional exclusion rules to ensure that it matches the clinical case definition. To implement these rules, the data engineer annotates the data with microbiology classification information of the UMLS metathesaurus [61]. This process could be improved with an expertise amplification system. The system should *summarize* data by showing the distribution of variables with linked brushing and filtering so that Lucy could see how the variable distributions are correlated. It could *guide* Lucy by suggesting correlated variables to the ones she selects. During validation of the cohort, Lucy should *interactively* be able to select data points to include. Finally, the system should be able to *accelerate* Lucy’s validation by suggesting exclusion rules based on her interactions.

After the cohort is finalized, Lucy faces a data cleaning task. The microbiology laboratory provides data for only a subset of antibiotics based on domain characteristics and institutional preferences. When using these data for predictive modeling, the unreported values must be filled by domain experts. To address this, we built Icarus [28] to amplify expertise in data augmentation. Icarus *guides* the domain expert by showing them high-impact data subsets for edits. It allows both direct *interactions* via edits and indirect *interactions* via rules. Finally, Icarus *accelerates* task completion by leveraging the UMLS classification to suggest general rules based on the domain expert’s single edit. It also allows the domain expert to preview the impact of a rule by *summarizing* the cells that will be impacted.

Owing to the subjective nature of this task, multiple domain experts need to come to consensus on unreported values. To amplify the consensus process, we designed Delphi [22], which visualizes the conflicts and redundancies in domain expert rules. It provides an overview of the data by visually *summarizing* the antibiotics and related rules in a node-link diagram. The node sizes *guide* the expert to regions of high conflict by encoding the number of data points affected. It allows domain experts to *interactively* edit the rule set by accepting and rejecting rules. Finally, it *accelerates* the domain experts’ task completion by automatically removing redundant rules after each edit.

Once domain experts have come to a consensus, the data set is ready for analysis. Our data scientist uses penalized logistic regression to model resistance [219]. During this stage, Lucy provides insights on the different variables and their relations. After model creation, Lucy can analyze and validate the results of the interactive analysis. For a given patient, the system should *summarize* its results by showing the probability of coverage along with confidence intervals. It should *guide* Lucy by drawing attention to any abnormal covariates whose value significantly deviates from others in the cohort. It should allow Lucy to *interactively* select covariates and rerun the model for a specific patient. It should *accelerate* the analysis by showing similar patients for whom the model should also be updated.

## Discussion

We have provided examples from the informatics literature to motivate the need for domain expert involvement in all steps of clinical data pipelines, from curation to analysis. Although this work is based on our experiences, we have done our best to do a targeted interdisciplinary review that can serve as a guide to clinical data projects. Our work is related to previous surveys in visual analytics in health care [188] and interactive systems [137]. Our survey is unique in that it provides a taxonomy on designing systems for amplifying expertise and focuses on the clinical data pipeline. Specifically, expertise amplification involves summarization, guidance, interactivity, and acceleration. Our case study illustrates how these can be applied to a clinical data pipeline.

### Conclusions

Effectively engaging domain experts is crucial for the success of data-driven workflows. We provide a novel framework for developing systems that amplify domain expertise. Amplification systems should summarize data, guide domain experts’ data navigation, allow domain experts to interact and update algorithms, and finally accelerate their task by learning from their interactions. This framework draws on research from multiple computer science disciplines. As we move toward data-driven workflows, interdisciplinary methods are necessary for the greatest impact. Empowering stakeholders to interact with the data directly can lead to faster and more impactful insights and decision making, which is vital for democratizing data to benefit society.

## Abbreviations

CDS: clinical decision support
EHR: electronic health record
HCI: human-computer interaction
NIH: National Institute of Health
UMLS: Unified Medical Language System
